# Prevalence of Mental Disorders, Cognitive Impairment, and Dementia Among Older Adults in Egypt: Protocol for a Systematic Review

**DOI:** 10.2196/14637

**Published:** 2020-07-24

**Authors:** Opeyemi Odejimi, George Tadros, Noha Sabry

**Affiliations:** 1 Psychiatric Liaison Department Birmingham and Solihull Mental Health Foundation Trust Birmingham United Kingdom; 2 Aston Medical School Aston University Birmingham United Kingdom; 3 Cairo Medical School Cairo University Cairo Egypt

**Keywords:** mental disorders, cognitive impairment, dementia, older adults, Egypt, prevalence, socio-demographic factors, systematic review, protocol

## Abstract

**Background:**

In Egypt, the population of older adults is rapidly growing. The last census in 2017 indicated that older adults numbered 94.8 million, which is a 2.56% increase from the 2006 census. There is growing evidence that the older population is at greater risk for some forms of mental disorders such as depression, dementia, and many more.

**Objective:**

This study aims to review the current evidence regarding the prevalence of mental disorders among older adults in Egypt. This will be achieved by estimating the current prevalence of mental disorders and identifying any sociodemographic correlations with mental disorders.

**Methods:**

An electronic search of 5 key databases (MEDLINE, PsycINFO, EMBASE, AMED, and PubMed) from their date of inception was conducted. In addition, scans of reference lists and searches of key journals, citations, and relevant internet resources were conducted. Studies were included if they were published in English, point prevalence studies, conducted with older Egyptians aged ≥60 years, and conducted using a validated diagnostic tool to ascertain mental disorders. Studies that did not meet any of these criteria were excluded.

**Results:**

This systematic review started in November 2018. The literature search of the 5 databases revealed 343 papers. After screening titles and abstracts, scanning citations and reference lists, and searching internet sources, a total of 38 full-text articles were accessed, of which 16 studies met the eligibility criteria and were included. We are currently in the process of data extraction and synthesis.

**Conclusions:**

This research will help bring the scale of mental disorders among older adults in Egypt to the forefront. This may help ensure evidence-based initiatives are established and that priority is given to resource allocation for geriatric mental health in Egypt.

**Trial Registration:**

PROSPERO International Prospective Register of Systematic Review CRD42018114831; https://www.crd.york.ac.uk/prospero/display_record.php?RecordID=114831

**International Registered Report Identifier (IRRID):**

DERR1-10.2196/14637

## Introduction

Egypt, like most developing countries, is facing a rising trend in mental disorders, with neuro-psychiatric disorders alone accounting for 19.8% of the burden of disability [1]. Egypt is central to the Arab world, an advantage that is supported by its geographical location, which spans from the northeastern part of Africa to the southwest region of Asia [2,3]. For instance, approximately 70% of the mental health professionals in Arab countries are Egyptians [4,5].

The 2006 census showed that the elderly population (≥60 years old) represents 7.2% of the total Egyptian population [6]. At the last census in 2017, adults ≥60 years old numbered 94.8 million, which is a 2.56% increase from the 2006 census [7]. The definition of old age varies in different countries and across various research studies, where it ranges from 55 years to 75 years. Old age in Egypt is believed to begin at 60 years, which is the age of retirement [8]. However, there have been proposals to change the retirement age from 60 years to 65 years, which might take effect by 2027 [9].

The older adult population in Egypt is mostly affected by noncommunicable diseases such as mental disorders [10]. Interestingly, mental disorders have always been recognized in Egypt; in fact, over 5 millennia ago, mental disorders were viewed as a physical ailment of the heart or uterus [11]. Moreover, one of the first three mental hospitals in the world was built in Cairo, Egypt [12]. There are 15 state psychiatric hospitals in Egypt, and nearly all medical schools in Egypt have a psychiatric department offering diploma, masters, and/or PhD degrees [2,12]. Currently, there are training programs for psychiatrists, psychiatric nurses, psychologists, and social workers; however, it is estimated that the psychiatrist to population ratio is 1:70,000 [2,13].

There are 3 main types of service providers in Egypt: public, private, and nongovernmental organizations [13]. Mental health services provided by the public sector are free; however, individuals who can afford private care would rather opt for this option due to the stigma associated with mental disorders and high quality of care offered [12]. Nongovernmental organizations are mostly outpatient services that usually have a sociopolitical or religious affiliation that influences the manner in which care and support are provided.

Mental disorders in Egypt have a strong cultural and religious influence. This is perhaps attributed to the strong family support, which is feasible among extended families. Thus, values, norms and beliefs about mental disorders are passed down from generation to generation. Several speculations have been held about the causes of mental disorders [3,12]. Some opinions about the causes include a personality weakness, laziness, exposure to a sudden fright, possession of evil spirits, use of magic, trauma to the head, heredity, emotional trauma, being looked at by evil eyes, and many more [12,14].

Mental disorder in Egypt is stigmatizing, and families are afraid of societal attitudes and discrimination, which often results in delays in seeking treatment [12,15]. In addition, views about mental disorders being genetic further heightens the discrimination and makes families hide this illness from others, thereby preventing shame, ridicule, and insult to the individual and the family as a whole [12].

A national survey of adults aged 18-64 years in 5 regions estimated the prevalence of mental disorders to be 16.93%, of which mood disorders, anxiety disorders, and multiple disorders were the most commonly identified [16]. Furthermore, a systematic review that focused on the prevalence of dementia in Egypt among individuals aged ≥50 years estimated a 2.01%-5.07% prevalence [17].

Currently, there are no registered studies that have investigated all mental disorders among the older adult population aged ≥60 years using the International Classification of Disease code 10, chapter 5 (F code) for mental and behavioral disorders in a single study. Some studies have estimated the prevalence of some mental disorders such as anxiety, depression, mixed anxiety and depressive disorders, mild cognitive impairment, or dementia [18-22].

Meanwhile, the profile of psychiatric presentation in Egypt reveals that individuals also present with obsessive-compulsive disorder, personality disorder, psychosis, schizophrenia, catatonia, substance misuse, suicide, and parasuicide [2,3]. However, little is being reported about the prevalence of these mental disorders among older adults. It is presumed that the cultural beliefs about an association between these mental disorders and moral failure, compounded by religious beliefs, further account for the significant under-presentation in mental health clinics [3,12]. Thus, there is little or no report of these illnesses among emerging studies.

Generally, it is reported that sociodemographic factors such as gender, educational status, employment status, and living arrangement strongly correlate with mental disorders in Egypt. It has been reported that being female, being unemployed, having little or no education, and living alone are associated with mental disorders among older adults (≥60 years) [18-22].

The needs of the older adult population in Egypt have long been ignored [2]. Moreover, Loza [13] pointed out that mental health received late recognition within health sector reform. Thus, the necessary development and financing were not initially a priority. Therefore, it can be inferred that mental disorders among older adults in Egypt have received very little attention. This systematic review will help identify all forms of mental disorders reported among older adults aged ≥60 years. Furthermore, it will estimate the current prevalence of these mental disorders and then identify the sociodemographic factors that correlate with mental health problems.

## Methods

### Reporting and Quality Assessment

This protocol adheres to the Preferred Reporting Items for Systematic Reviews and Meta-Analyses (PRISMA) 2009 checklist and is documented on the PROSPERO database (PROSPERO registration number: CRD42018114831).

PRISMA has been chosen to facilitate transparent reporting of this systematic review. We acknowledge that PRISMA has some shortcomings, such as not being able to gauge the quality of included papers [23]. This is the reason we are conducting a quality assessment of the included papers. We are using the Newcastle-Ottawa scale adapted for cross-sectional studies to assess the quality of the included studies. In addition, we are clearly reporting this protocol to allow others to judge and easily replicate this review [23].

### Data Sources and Searches

A search was conducted in 5 databases: MEDLINE, PsycINFO, EMBASE, AMED, and PubMed. Databases were searched from their date of inception in order to compare the trend of mental disorders over the years. Both medical subject headings and free-text words (searches of titles and abstract) were carried out in the electronic databases. Searches included terms relating to or describing mental health disorders, cognitive impairment, dementia, prevalence, older adult, and Egypt. Boolean operators, truncation, and combinations of AND, OR, and ADJ were used when necessary. A sample of combined search terms in an electronic database is shown in Textbox 1.

In addition, we searched the reference lists of all studies that met the inclusion criteria and relevant systematic reviews for any potential eligible studies. We also searched key journals, citations, and relevant internet resources to ensure this review contains all possible studies and to ascertain that studies not indexed in the 5 chosen databases are included in this review. We contacted authors if additional information was required.

**Sample of combined search terms used in an electronic database.**

EMBASE

(((“MENTAL DISEASE”/ OR (mental ADJ1 (disorder* OR disease* OR illness*)).ti,ab OR (Psychiatr* ADJ (disorder* OR disease* OR illness*)).ti,ab OR DEMENTIA/ OR “COGNITIVE DEFECT”/ OR (“cognitive impairment*” OR “cognitive defect*” OR “cognitive dysfunction”).ti,ab OR (dementia).ti,ab OR PSYCHIATRY/) AND ((elder* OR “old* adult*” OR geriatric* OR “old* person” OR “old* people” OR “old* age” OR “age* people” OR “aging people” OR “age* person” OR “aging person” OR “old* patient*” OR psychogeriatric* OR “aging patient*” OR “age* patient*”).ti,ab OR AGED/ OR GERIATRICS/)) AND (EPIDEMIOLOGY/ OR “CROSS-SECTIONAL STUDY”/ OR “HEALTH SURVEY”/ OR (survey* OR epidemiolog* OR cross-sectional OR prevalence*).ti,ab)) AND ((Egypt*).ti,ab OR EGYPT/)

### Inclusion and Exclusion Criteria

Studies conducted in any setting such as hospitals (both inpatients and outpatients), residential homes, household surveys, and others within Egypt were included in this review. Studies were included if they were published in English, point prevalence studies, conducted with older Egyptians aged ≥60 years, and conducted with a validated diagnostic tool to ascertain mental disorders. Validation of diagnostic tools and prevalence rate is based on self-reported declaration of the original authors of the included papers. Studies that did not meet any of the criteria were excluded. It should be noted that point prevalence is used as recommended by Streiner et al [24].

### Data Extraction

Two review authors (OO and GT) independently screened the studies retrieved from all sources to identify those that potentially met the inclusion criteria. The full text of these potentially eligible studies was retrieved and independently assessed for eligibility. If there was any disagreement between the two authors, this was resolved through discussion and, if necessary, with a third member (NS) for mediation. A piloted data extraction sheet was used to extract data from the included studies. Extracted information included author’s name, year of publication, study setting(s), city, duration of the study, demographics, number of participants, method of recruitment, diagnostic test used, mental disorders studied, prevalence of mental disorders, and existence of comorbidities, current medication, and smoking status.

### Quality Assessment

The Herzog modification of the Newcastle-Ottawa scale adapted for cross-sectional studies was used to assess the quality of the included studies. The scale assesses 3 main domains: selection, comparability, and outcomes. Each study could attain a maximum of 10 points. Studies with 0-4 points were classed as unsatisfactory, 5-6 points as satisfactory, 7-8 points as good, and 9-10 points as very good. Two review authors (OO and GT) assessed the risk of bias, and any disagreements were resolved by discussion and, if necessary, with a third member (NS) for mediation.

### Data Analysis and Synthesis

A descriptive tabular summary of included studies was documented, categorizing details based on study setting(s), city, duration of the study, demographics, number of participants, method of recruitment, diagnostic test used, mental disorders studied, and prevalence of mental disorders. The quality or risk of bias of the included studies was also evaluated.

Preliminary synthesis of the sample and population as well as demographic information (such as age, gender, education, employment, residency, marital status, and living arrangement) was carried out. Furthermore, the correlation between mental disorders and sociodemographic details within and between studies will be explored. In addition, the robustness of the synthesis will be assessed based on the methodological quality of the studies. A narrative synthesis of the findings of included studies will be provided. This is because we anticipate that the included studies will be methodologically diverse.

## Results

**Figure 1:**
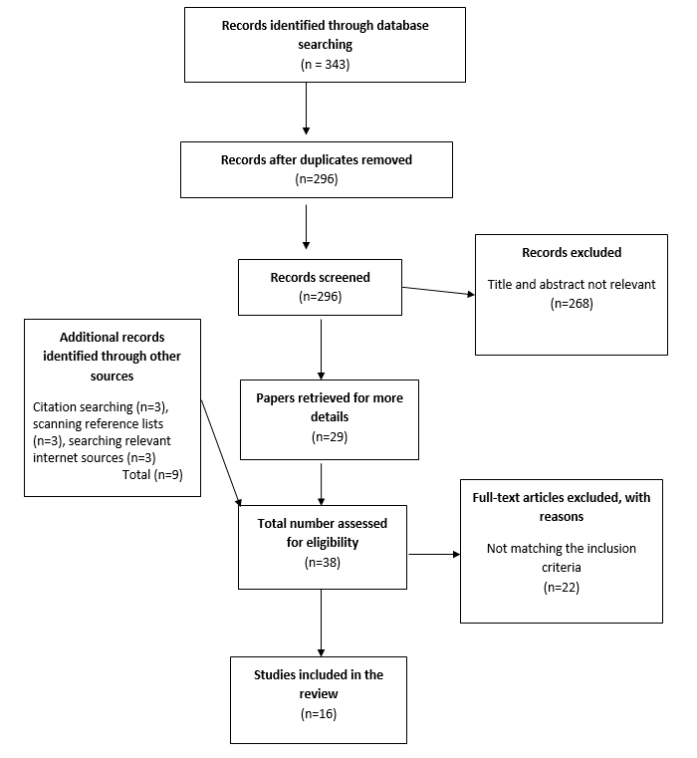
Preferred Reporting Items for Systematic Reviews and Meta-Analyses (PRISMA) flow illustrating the search outcomes.

This systematic review started on November 1, 2018. The search has been completed. The literature search of the 5 databases revealed 343 papers. After screening titles and abstracts, scanning the citations and reference lists, and searching internet sources, a total of 38 full-text articles were accessed, of which 16 studies met the eligibility criteria and were included. Figure 1 provides the details of the search outcome.

All identified studies carried out point prevalence studies ranging from a period of 3 months to 2 years with adults aged ≥60 years. We are currently in the process of data extraction and synthesis. The findings of this review may help bring the scale of mental disorders among older adults in Egypt to the forefront, which may result in evidence-based initiatives being established as well as prioritized resource allocation for geriatric mental health in Egypt. The results of this systematic review will be published in a peer-reviewed journal.

## Discussion

This systematic review identified 16 studies matching the eligibility criteria. This study is conducted as the initial step of a longitudinal study on mental disorders among the older adult population in Egypt. The paucity of existing studies highlights the need for further research.

This review will help identify mental disorders among older adults. It will also describe any associations between mental disorders and sociodemographic status. The findings may help bring the impact of mental health problems among older adults in Egypt to the forefront for policy makers, service providers, and researchers.

There might be a need to develop a uniformed approach to the demographic and social determinants of mental disorders in older adults in Egypt and similar countries. This research could also serve as a guide when developing interventions and prioritizing resource allocation for mental health in Egypt.

Regarding potential limitations, it is anticipated that some potentially relevant studies may not be included due to selecting studies only published in English.

## Abbreviations

PRISMA: Preferred Reporting Items for Systematic Reviews and Meta-Analyses.
